# Criterion validity assessment of the 24-hour movement behaviors questionnaire (24HMBQ) for Chinese college students: validation study

**DOI:** 10.7717/peerj.21490

**Published:** 2026-07-28

**Authors:** Teck Cheng Tan, Jiaxin Zheng, Tianle Chen, Yiting Liu, Yan Luo, Yanxiang Yang, Tao Huang

**Affiliations:** 1 School of Exercise and Health, Shanghai University of Sport, Shanghai, China; 2 School of Physical Education and Sport Science, Fujian Normal University, Fuzhou, China; 3 Department of Physical Education, Shanghai Jiao Tong University, Shanghai, China

**Keywords:** 24-hour movement behavior, Criterion validity, Accelerometer, Questionnaire, College students

## Abstract

**Background:**

Accumulating studies have investigated the combined effects of 24-h movement behaviors on physical and mental health. The 24-hour Movement Behaviors Questionnaire (24HMBQ) was recently developed to measure the 24-hour movement behaviors in college students. However, its criterion validity remains to be elucidated. This study aimed to investigate the criterion validity of the 24HMBQ against accelerometer-derived data among Chinese college students.

**Methods:**

Eighty-eight college students (age = 20.2 ± 2.2 years) participated in this study. The Axivity AX3 accelerometers were used to objectively measure physical activity (PA), sedentary behavior (SB), and sleep levels over a consecutive seven-day period. At the last day, participants completed the 24HMBQ to self-report their PA, SB, and sleep levels. Paired-sample *t*-test, Spearman’s rank correlation test, and Bland-Altman plot were used to examine the degree of agreement between 24HMBQ and accelerometer data.

**Results:**

Spearman’s correlation coefficients indicated strong agreement for sleep metrics (Rho = 0.77, *p* < 0.001 for weekdays; Rho = 0.68, *p* < 0.001 for weekends) and moderate agreement for SB (Rho = 0.55, *p* < 0.001 for weekdays; Rho = 0.33, *p* < 0.01 for weekends) between 24HMBQ and accelerometer measurements. However, lower correlations were observed for physical activities including light PA (Rho = 0.30, *p* < 0.01) and moderate-to-vigorous PA (MVPA; Rho = 0.15, *p* > 0.05).

**Conclusion:**

The 24HMBQ showed good criterion validity for sleep and moderate validity for sedentary behavior, but limited validity for physical activity (especially MVPA).

## Introduction

Physical activity (PA), sedentary behavior (SB) and sleep constitute the movements within a 24-hour period, which are collectively referred to as 24-hour movement behavior (Rollo, Antsygina & Tremblay, 2020; Rosenberger et al., 2019). Studies have shown that optimizing the allocation of time across 24-hour movement behaviors is associated with better physical and mental health outcomes, including reduced chronic disease risk, improved mental well-being, and enhanced quality of life (Feng et al., 2021; Tremblay et al., 2016; Walsh et al., 2018). In response to this growing body of research, several countries, such as Canada and Australia, have developed 24-hour movement guidelines to support public health initiatives and behavioral recommendations across the lifespan (Rollo, Antsygina & Tremblay, 2020; Rosenberger et al., 2019; Tremblay et al., 2016).

Currently, accumulating studies have focused on examining the associations between adherence to 24-hour movement behaviors and various health outcomes. To measure 24-hour movement behaviors, researchers predominantly rely on wearable device (*e.g.*, accelerometers) (Clarke & Janssen, 2021; Duncan et al., 2018) or self-reports (*e.g.*, questionnaires and activity log) (Hidding et al., 2020; Peddie, Scott & Haszard, 2021; Song et al., 2021). Accelerometers provide detailed and objective estimates of duration of PA, SB and sleep (Vähä-Ypyä et al., 2015). However, the use of accelerometers in large-scale studies faces challenges due to high costs and potential compliance issues (Željko & Adrian, 2015). Conversely, subjective self-report methods are often favored for their simplicity and low cost (Sallis & Saelens, 2000).

College students often prioritize their studies and online activities over PA, leading to an increase in SB (Castro et al., 2020). The PA guidelines for adults suggest a minimum of 150 min of moderate-to-vigorous PA (MVPA) or 75 min of vigorous-intensity PA (VPA) each week (Bull et al., 2020). However, recent studies indicate that college students typically do not meet these recommendations (Babaeer et al., 2022), and shows poor sleep quality and duration (Memon et al., 2021). Valid and reliable measurements are crucial to fully understand the impact of the entire 24-hour movement behaviors on health outcomes among college students.

Given this context, the 24-hour Movement Behaviors Questionnaire (24HMBQ) was recently developed to measure the 24-hour movement behaviors in college students (Zheng et al., 2023). The 24HMBQ was specifically developed for Chinese college students to capture their daily activity patterns and provide a comprehensive assessment of 24-hour movement behaviors in this population. It encompasses three dimensions, including sleep, SB (*i.e.,* various sedentary activities like studying and leisure), and PA (*i.e.,* a range of activities at different intensities such as daily exercise, daily transportation, and daily dormitory life). Initial validation of the 24HMBQ among Chinese college students has shown promising results concerning the face validity, content validity, convergent validity, and test-retest reliability (Zheng et al., 2023). However, its criterion validity remains unknown. Prior studies have suggested that subjective self-reports often differ significantly from objective measures using devices such as accelerometers(Arumugam et al., 2023; Wang, Chen & Zhuang, 2013), highlighting the importance to further assess the criterion validity of the 24HMBQ in comparison with accelerometer data. Therefore, the aim of this study was to assess the criterion validity of the 24HMBQ among Chinese college students.

## Methods

### Participants

We employed a convenience sampling method and recruited participants through social media posts and physical education classes at a university in Shanghai, China. Inclusion criterion was college students who were 18-35 years old. Participants were excluded if they: (a) had physical and mental conditions preventing them from participating in PA; (b) were not willing to wear an accelerometer and fill out the 24HMBQ; or (c) had sleep disorders and mental disorders. These exclusion criteria were assessed *via* a brief baseline self-report screening questionnaire and were not based on clinical diagnosis.

Initially, 144 college students were reached. During the study, 29 participants withdrew for various reasons. Of the remaining 115 participants, 27 were excluded from analysis due to not meeting the accelerometer wear-time criteria (*i.e.,* at least three days including two weekdays and one weekend day with a minimum of 16 h per day). Consequently, 88 participants were included in the final analyses.

### Procedure

The research was conducted in accordance with the Declaration of Helsinki. Ethical approval for the study was obtained from the Institutional Review Board of the authors’ affiliated university (approval code: H2022225I). The aim of the study was explained to all participants prior to data collection. They were assured that their information would remain confidential and informed that participation was voluntary, with the right to withdraw at any time. Written informed consent was obtained from each participant.

### Instruments and data processing

#### 24HMBQ

The 24HMBQ for Chinese college students is a newly developed instrument that comprehensively assesses sleep, SB, and PAs (Zheng et al., 2023). The 24HMBQ consists of 24 items in three sections, namely sleep, SB, and PA sections.

The sleep section consists of six items focusing on students’ sleep habits, measuring details such as usual bedtime and wake-up times on weekdays and weekends. Items such as “During the past week, what time did you usually wake up in the morning?” and “During the past week, how much time did you usually spend taking a nap during the day?” are designed to capture nighttime sleep and daytime napping patterns, respectively. The total daily sleep duration (minutes per day (min/day)) can be therefore calculated by quantifying the interval between bedtime and wake-up time, plus the duration of daytime napping.

The SB section consists of eight items assessing students’ time spent in various sedentary activities. The average daily duration of seated activities for study or work purposes, leisure time on electronic devices, and other inactive activities such as eating or transportation were asked. For example, “During the past week, on average, how much time per day did you spend on electronic screen-based devices for entertainment while sitting or lying down?” The total time spent in SBs on weekdays and weekends is calculated by summing the durations reported in these questions, with data expressed in minutes per day (min/day).

The PA section includes ten items concerning students’ involvement in PAs of varying intensities, such as daily exercises, transportation, and dormitory life activities. Item like “During the past week, how often and for how long did you engage in vigorous-intensity physical activities?” provides examples to clarify intensity levels, including “fast bicycling” for vigorous or “brisk walking” for moderate activities. The total time spent in PAs of different intensities over a week is calculated and reported in minutes per day (min/day).

#### Accelerometer

On the first day of data collection, each participant was issued an Axivity AX3 tri-axial accelerometer (Axivity Ltd., Newcastle, UK) (Doherty et al., 2017) and instructed to wear it continuously for seven consecutive days to objectively collect PA, SB, and sleep data. The Axivity AX3 has demonstrated excellent agreement with other research-grade wrist-worn accelerometers for average acceleration (ICC = 0.95, 95% CI [0.87–0.98]) (Rowlands et al., 2018). Participants wore the devices on their non-dominant wrist and were instructed to remove them only for water-related activities (*e.g.*, bathing or swimming). Participants were required to record any instances of non-wear time to ensure data accuracy.

The Axivity AX3 devices were initialized using the open-source software OmGui (Open Movement, Newcastle University, UK). The accelerometer was configured to record tri-axial acceleration data at a sampling rate of 100 Hz, within a dynamic range of ±8g. Raw data were downloaded in .cwa format for processing and analysis. Acceptance criteria for valid wearing time were set at least three days (two weekdays and one weekend day) with at least 16 h of wear time per day (Trost, McIver & Pate, 2005). Non-wear time was precisely identified as any 60-minute interval with a standard deviation of acceleration below 13.0 mg (Sabia et al., 2014). Participants who did not meet these wear-time criteria were excluded from the analyses.

The Axivity AX3 raw data were processed with R-based GGIR package (version 3.0-0) (Migueles et al., 2019). We evaluated raw acceleration data using the Euclidean Norm Minus One (ENMO) metric, computing average levels every five seconds and adjusting negative values to zero. This choice of the ENMO metric was based on its established efficacy in accurately reflecting PA patterns and energy expenditure, while also simplifying data standardization and comparison. In this study, sleep analysis focused on identifying the start and end of sleep within the Sleep Period Time (SPT) window using accelerometer data. This process involved detecting periods of rest marked by inactivity to measure sleep duration accurately (Van Hees et al., 2015). PA analysis involved segmenting accelerometer data into intensity levels using specific cut points to distinguish between inactivity, light PA (LPA), moderate PA (MPA), and vigorous PA (VPA) levels. Cut points were established to differentiate activity levels: LPA at 45–99 mg, MPA at 100–429 mg, and VPA exceeding 430 mg. These thresholds were adopted from validated studies on adult and young-adult populations, which have demonstrated acceptable validity and reliability in estimating activity intensity among college-aged participants (Hildebrand et al., 2017; Migueles et al., 2017). This approach facilitated a detailed analysis of the participants’ SB and PAs, allowing for nuanced distinctions between varying levels of activity intensity. This refined method has helped in accurately differentiating between periods of non-wear and genuine SB.

If participants had fewer than seven valid days of data, the mean daily values were calculated based on available valid days. No data imputation was performed. To align with the 24HMBQ recall period, a 5:2 weekday-to-weekend weighting was applied to compute weekly average estimates of PA, SB, and sleep. As part of data quality control, we conducted range checks on accelerometer-derived movement-behaviour estimates; values outside feasible daily ranges were verified and excluded if clearly erroneous.

### Anthropometrics and demographics

Body mass, height and other demographic information were self-reported *via* a questionnaire.

### Statistical analysis

The IBM SPSS Statistics 23.0 software (IBM Corp., Armonk, NY, USA) was used for statistical analyses. Differences between measurements obtained from the 24HMBQ and the Axivity AX3 were evaluated using paired t-tests. The correlations between 24HMBQ and Axivity AX3 estimates were examined by the Spearman rank correlation test. The strength of the Spearman correlation (Rho) was categorized based on the following criteria: weak (<0.30), low (0.30–0.49), moderate (0.50–0.69), strong (0.70–0.89), and very strong (≥0.90) (Hinkle, Wiersma & Jurs, 2003). The mean difference and limits of agreement (LOA) recommended by Bland and Altman were used to evaluate the agreement between 24HMBQ and Axivity AX3 (Bland & Altman, 1986). The mean difference was determined by averaging the differences between 24HMBQ and Axivity AX3 (subtracting the latter from the former), representing the average difference between the two measurements. The LOA was derived by adding and subtracting 2 × the standard deviation of the difference to the mean difference, denoting the potential range of difference between the two methods. The Bland-Altman plot was used to graphically present the degree of agreement between 24HMBQ and Axivity AX3.

## Results

Table 1 summarizes the demographic information of the participants. The average height was 170.6 ± 9.0 cm, and the mean body weight was 69.1 ± 11.8 kg. The average body mass index (BMI) was 21.2 ± 3.0 kg/m^2^. The majority of the participants were undergraduate students (78.41%, *n* = 69), while graduate students accounted for 21.59% (*n* = 19). A substantial proportion of the participants were enrolled in natural science majors (70.45%, *n* = 62), whereas 29.55% (*n* = 26) of the participants pursued majors in the social sciences and humanities.

**Table 1:** Demographic characteristics of study participants.

Variables	Males (*n* = 42)	Females (*n* = 46)	All (*n* = 88)
Age (Years)	19.8 ± 1.8	20.6 ± 2.5	20.2 ± 2.2
Height (cm)	177 ± 5.8	164.8 ± 9.0	170.6 ± 9.0
Body mass (kg)	69.1 ± 11.8	55.7 ± 9.0	62.1 ± 12.4
BMI (kg/m^2^)	22.0 ± 3.3	20.4 ± 2.5	21.2 ± 3.0
Major			
*Natural Sciences*	38 (90.48%)	24 (52.17%)	62 (70.45%)
*Social Sciences and Humanities*	4 (9.52%)	22 (47.83%)	26 (29.55%)
Education level			
*Undergraduate student*	37 (88.10%)	32 (69.57%)	69 (78.41%)
*Graduate student*	5 (11.90%)	14 (30.43%)	19 (21.59%)

**Notes.**

Data are presented as Mean ± SD or numbers (%).

Table 2 shows the differences and correlations between 24HMBQ estimates and Axivity AX3 accelerometer data. For sleep, the correlations were strong (weekday Rho = 0.77, *p* < 0.001; weekend Rho = 0.68, *p* < 0.001). For SB, correlations were moderate on weekdays (Rho = 0.55, *p* < 0.001) and low on weekends (Rho = 0.33, *p* < 0.01). Regarding PA, the Rho for LPA was 0.30 (*p* < 0.01) and the Rho for MVPA was 0.15 (*p* > 0.05).

**Table 2:** Paired *t*-test and Spearman’s correlation between the 24HMBQ and Axivity AX3 (*n* = 88).

Movement Behavior	24HMBQ	Axivity AX3	t (df)	Spearman’s Rho
Weekday Sleep (min/day)	439.52 ± 57.16	444.07 ± 44.80	−1.221(87)	0.77^***^
Weekend Sleep (min/day)	495.59 ± 75.24	480.30 ± 67.03	2.668(87)^**^	0.68^***^
Weekday SB (min/day)	717.95 ± 146.53	736.14 ± 80.89	−1.374(87)	0.55^***^
Weekend SB (min/day)	709.20 ± 170.96	700.38 ± 85.75	.511(87)	0.33^**^
LPA (min/day)	47.06 ± 44.51	73.37 ± 23.86	−5.979(87)^**^	0.30^**^
MVPA (min/day)	50.90 ± 46.31	64.48 ± 24.74	−2.594(87)^**^	0.15

**Notes.**

* *p* < 0.05.

** *p* < 0.01.

*** *p* < 0.001

Table 3 presents the results of Bland-Altman analysis, which evaluates the agreement between 24HMBQ and Axivity AX3 data. The mean differences in sleep duration for weekdays and weekends were −4.55 minutes/day (95% CI [−12.00 to 2.90]) and 15.29 minutes/day (95% CI [3.84 to 26.75]), respectively. SB showed a mean difference of −18.19 minutes/day for weekdays (95% CI [−44.65 to 8.27]) and 8.83 minutes/day for weekends (95% CI [−25.71 to 43.37]), respectively. For PA, the mean differences were -26.32 minutes/day (95% CI [−35.11 to −17.52]) for LPA and -13.58 minutes/day (95% CI [−24.04 to −3.12]) for MVPA, respectively. The LOA varied across the three movement behaviors (Table 3).

**Table 3:** Bland–Altman analysis results for Movement Behaviors.

Behaviors	Mean difference (95% CI)	Limits of agreement	
		Lower limit (95% CI)	Upper limit (95% CI)
Weekday Sleep (min/day)	−4.55 (−12.00, 2.90)	−74.50 (−87.41, −61.59)	65.4 (52.49, 78.31)
Weekend Sleep (min/day)	15.29 (3.84, 26.75)	−92.24 (−112.08, −72.39)	122.82 (102.98, 142.67)
Weekday SB (min/day)	−18.19 (−44.65, 8.27)	−266.52 (−312.34, −220.69)	230.13 (184.31, 275.96)
Weekend SB (min/day)	8.83 (−25.71, 43.37)	−315.36 (−375.18, −255.53)	333.01 (273.19, 392.84)
LPA in one week (min/day)	−26.32 (−35.11, −17.52)	−108.89 (−124.13, −93.65)	56.26 (41.02, 71.50)
MVPA in one week (min/day)	−13.58 (−24.04, −3.12)	−111.79 (−129.92, −93.67)	84.63 (66.51, 102.76)

**Figure 1:**
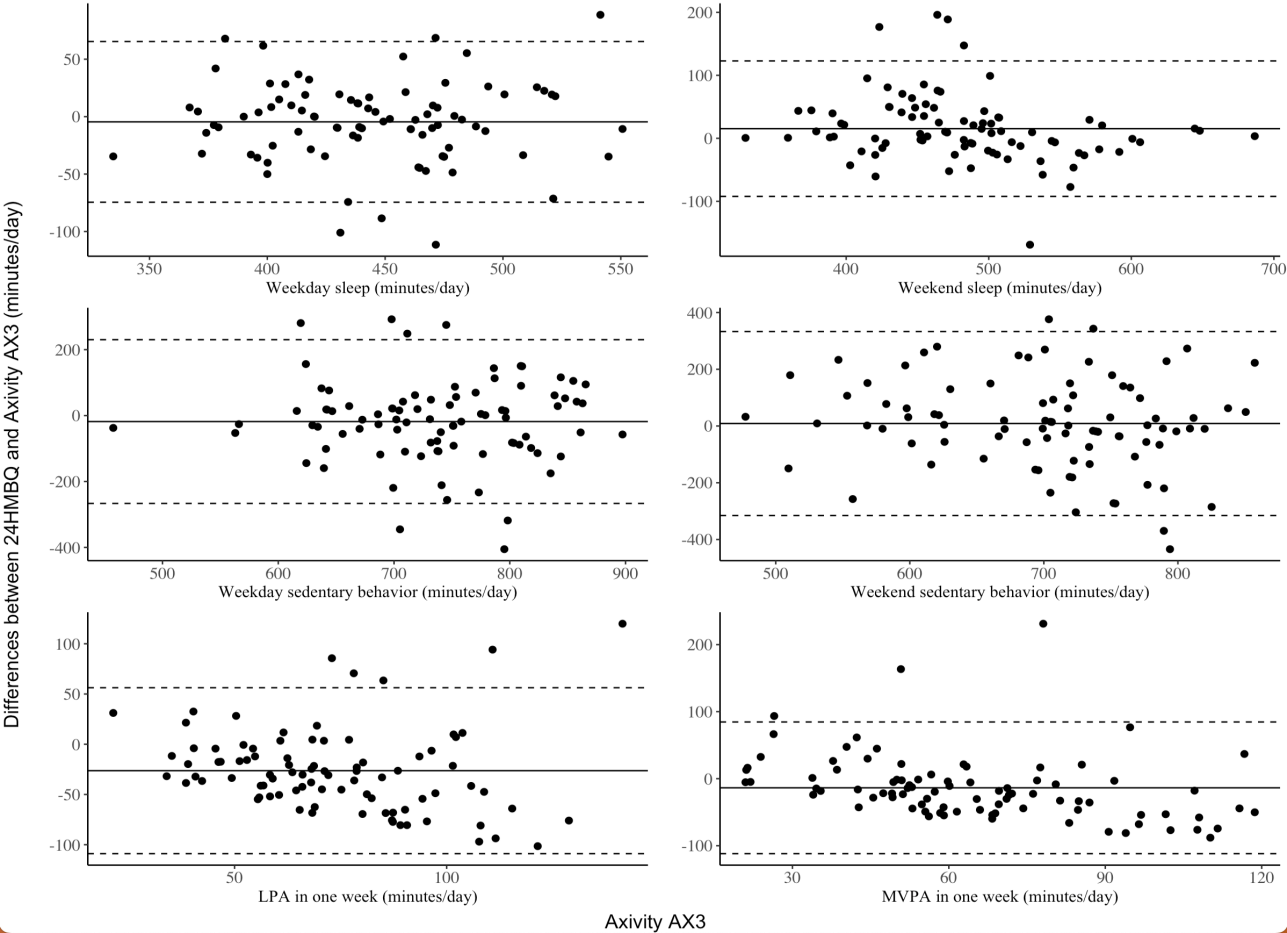
Bland-Altman plots for agreement analysis between 24HMBQ and Axivity AX3.

Figure 1 depicts the Bland-Altman plots, which illustrate the differences (24HMBQ minus Axivity AX3) against the measurements from Axivity AX3. Each plot displays the mean difference between 24HMBQ and Axivity AX3 as a solid horizontal line. LOAs are illustrated with two dashed horizontal lines, situated below and above the mean difference line, respectively. Mean bias was small for some outcomes; however, the limits of agreement were wide, indicating substantial individual-level variability between the 24HMBQ and accelerometer estimates. Additionally, the residual plots for weekday sleep, weekend sleep, weekday sedentary behavior, and weekend sedentary behavior showed a random distribution around the zero line, indicating no systematic bias and no obvious linear trends in these plots. However, the slight downward linear trends for LPA and MVPA suggest that as the values of LPA and MVPA increase, the 24HMBQ tends to increasingly underestimate these measures.

Overall, Bland–Altman analyses indicated small-to-moderate mean biases across domains but wide limits of agreement, suggesting limited agreement at the individual level. The downward trends observed for LPA and MVPA indicate increasing underestimation by the 24HMBQ at higher activity levels. Larger negative biases and wider limits of agreement were observed for MVPA, suggesting greater variability across individuals for higher-intensity physical activity estimates.

## Discussion

This study assessed the criterion validity of the 24HMBQ against wrist-worn accelerometer estimates in Chinese college students. The results indicated stronger validity for sleep duration, moderate performance for sedentary behavior, and limited validity for PAs, particularly MVPA.

Regarding the sleep estimates, the results showed a relatively strong correlation and good agreement for both weekday sleep and weekend sleep between the 24HMBQ and Axivity AX3 estimates. The findings suggest that 24HMBQ is a valid instrument to assess sleep duration among college students. Overall, the current results are in line with some previous studies. For instance, a previous study observed a correlation (*r* = 0.66) between the Daily Activity Behaviours Questionnaire (DABQ) and activPAL4 accelerometer estimates for weekday sleep and in working adults (Kastelic et al., 2022). In contrast, Alkhraiji, Barker & Williams (2022) observed weak correlations between the Global Student Health Survey (GSHS) and GENEActiv accelerometer estimates of sleep in adolescents. This comparison pertains to sleep duration derived from the accelerometer SPT-window approach rather than sleep architecture (*e.g.*, REM or deep sleep), which was beyond the scope of this study (Van Hees et al., 2015).

For the SB estimates, our analysis revealed moderate correlations and good agreements between the 24HMBQ and Axivity AX3 estimates of total SB. A recent systematic review and meta-analysis showed that single-item self-reported measures of SB tend to underestimate sedentary time compared to accelerometer estimates (Prince et al., 2020). However, the 24HMBQ collects the duration of engagement in specific SB types (*i.e.,* study, work, electronic screen-based devices for entertainment) by six items. Similarly, the study by Keadle et al. assessed the validity of Activities Completed over Time in 24 Hours (ACT24), a web-based previous-day recall instrument, and found a correlation of *R* = 0.61 for overall SB time (including time on personal care, leisure time, work, shopping/errands, household tasks, and transport) compared to accelerometer estimates (Keadle et al., 2023). Together with our results, the evidence indicates that questionnaires assessing different types of SB with multiple items may provide more accurate estimates than single-item questionnaires. Notably, correlations were weaker on weekends than weekdays, suggesting greater variability in recalling weekend sedentary patterns (Nusser et al., 2012). In addition, the wide limits of agreement indicate that while group-level SB estimates may be acceptable, individual-level interchangeability with accelerometry is limited (Bland & Altman, 1986).

Relative to sleep and sedentary behaviour, validity for physical activity was limited. Correlations were low for LPA and weak for MVPA, and Bland–Altman analyses suggested increasing underestimation at higher activity levels with wide limits of agreement. These findings indicate that 24HMBQ-derived PA estimates, particularly MVPA estimates, should be interpreted cautiously, and that accelerometer-based methods remain preferable when precise PA quantification is required. However, the findings are in agreement with previous validation studies. The existing validation studies for a variety of PA questionnaires also demonstrated a weak to moderate agreement between the questionnaire and device estimates in both adults and youth (Ács et al., 2020; Alkahtani, 2016; Lee et al., 2020; Wang, Chen & Zhuang, 2013). The lower accuracy of PA estimations might be explained by a couple of reasons. First, individuals, particularly those with limited exercise experience, might find it challenging to evaluate the PA intensity, thereby leading to significant deviations in PA estimates (Canning et al., 2014). Second, the questionnaire can assess domain-specific PA, including PA in leisure time, commuting, household, etc. Therefore, the responders may be subject to limitations in recall bias and may neglect some PAs (Cerin et al., 2016). Agreement may also vary with accelerometer processing decisions and the cut-points used to classify intensity, which can particularly affect MVPA estimates (Hildebrand et al., 2017; Migueles et al., 2017).

This study has several limitations. First, the recall bias might lead to errors in self-report of 24-hour movement behaviors, therefore the possibility for over or under estimation cannot be ruled out. Second, this study used a single-university convenience sample with a modest sample size (*n* = 88), which may limit the precision of validity estimates (particularly for weaker correlations) and reduce generalizability to broader college student populations. Sample composition (*e.g.*, majors and sex distribution) may also influence movement patterns and reporting accuracy. Third, the sample size limited our ability to conduct adequately powered subgroup analyses (*e.g.*, by sex). Finally, although accelerometers are widely used criterion measures, some activities (*e.g.*, cycling, swimming, and other water-based exercise) may be underestimated or not well captured by wrist-worn devices.

Despite these limitations, the present study provides preliminary evidence supporting the 24HMBQ as a feasible and low-cost tool for assessing 24-hour movement behaviors in college students. Future research should focus on refining the questionnaire items related to physical activity intensity and domain-specific contexts to enhance measurement accuracy. Additionally, validation in other age groups and cultural contexts would help extend the generalizability and applicability of the 24HMBQ in public health surveillance and behavioral monitoring.

## Conclusion

This study assessed the criterion validity of the 24HMBQ against accelerometer-derived estimates in Chinese college students. The 24HMBQ showed good validity for sleep duration and moderate validity for sedentary behavior, but limited validity for physical activity, particularly MVPA; therefore, it may be appropriate for group-level surveillance but should be interpreted cautiously for individual-level PA quantification.

## Supplemental Information

10.7717/peerj.21490/supp-1 Supplemental Information 1 Raw data.

## Abbreviations

PA: Physical activity
SB: Sedentary behavior
24HMBQ: 24-Hour Movement Behavior Questionnaire
SD: Standard deviation
LOA: limits of agreement
LPA: low-intensity physical activity
MPA: moderate physical activity
VPA: vigorous-intensity physical activity
MVPA: moderate-to-vigorous-intensity physical activity

## Additional Information and Declarations

### Data Availability

The following information was supplied regarding data availability: The raw data is available in the Supplemental Files.

## References

[ref-1] Ács P, Betlehem J, Oláh A, Bergier J, Melczer C, Prémusz V, Makai A (2020). Measurement of public health benefits of physical activity: validity and reliability study of the international physical activity questionnaire in Hungary. BMC Public Health.

[ref-2] Alkahtani SA (2016). Convergent validity: agreement between accelerometry and the Global Physical Activity Questionnaire in college-age Saudi men. BMC Research Notes.

[ref-3] Alkhraiji MH, Barker AR, Williams CA (2022). Reliability and validity of using the global school-based student health survey to assess 24 h movement behaviours in adolescents from Saudi Arabia. Journal of Sports Sciences.

[ref-4] Arumugam A, Mohammad Zadeh SA, Zabin ZA, Hawarneh TME, Ahmed HI, Jauhari FS, Alkalih HY, Shousha TM, Moustafa IM, Häger CK (2023). Sedentary and physical activity time differs between self-reported ATLS-2 physical activity questionnaire and accelerometer measurements in adolescents and young adults in the United Arab Emirates. BMC Public Health.

[ref-5] Babaeer L, Stylianou M, Leveritt M, Gomersall S (2022). Physical activity, sedentary behavior and educational outcomes in university students: a systematic review. Journal of the American College of Health.

[ref-6] Bland JM, Altman DG (1986). Statistical methods for assessing agreement between two methods of clinical measurement. Lancet.

[ref-7] Bull FC, Al-Ansari SS, Biddle S, Borodulin K, Buman MP, Cardon G, Carty C, Chaput J-P, Chastin S, Chou R, Dempsey PC, DiPietro L, Ekelund U, Firth J, Friedenreich CM, Garcia L, Gichu M, Jago R, Katzmarzyk PT, Lambert E, Leitzmann M, Milton K, Ortega FB, Ranasinghe C, Stamatakis E, Tiedemann A, Troiano RP, Van der Ploeg HP, Wari V, Willumsen JF (2020). World Health Organization 2020 guidelines on physical activity and sedentary behaviour. British Journal of Sports Medicine.

[ref-8] Canning KL, Brown RE, Jamnik VK, Salmon A, Ardern CI, Kuk JL (2014). Individuals underestimate moderate and vigorous intensity physical activity. PLOS ONE.

[ref-9] Castro O, Bennie J, Vergeer I, Bosselut G, Biddle SJH (2020). How sedentary are university students? A systematic review and meta-analysis. Prevention Science.

[ref-10] Cerin E, Cain KL, Oyeyemi AL, Owen N, Conway TL, Cochrane T, D VAND, Schipperijn J, Mitáš J, Toftager M, Aguinaga-Ontoso I, Sallis JF (2016). Correlates of agreement between accelerometry and self-reported physical activity. Medicine and Science in Sports and Exercise.

[ref-11] Clarke AE, Janssen I (2021). A compositional analysis of time spent in sleep, sedentary behaviour and physical activity with all-cause mortality risk. International Journal of Behavioral Nutrition and Physical Activity.

[ref-12] Doherty A, Jackson D, Hammerla N, Plötz T, Olivier P, Granat MH, White T, Van Hees VT, Trenell MI, Owen CG, Preece SJ, Gillions R, Sheard S, Peakman T, Brage S, Wareham NJ (2017). Large scale population assessment of physical activity using wrist worn accelerometers: the UK biobank study. PLOS ONE.

[ref-13] Duncan S, Stewart T, Mackay L, Neville J, Narayanan A, Walker C, Berry S, Morton S (2018). Wear-time compliance with a dual-accelerometer system for capturing 24-h behavioural profiles in children and adults. International Journal of Environmental Research and Public Health.

[ref-14] Feng J, Zheng C, Sit CH-P, Reilly JJ, Huang WY (2021). Associations between meeting 24-hour movement guidelines and health in the early years: a systematic review and meta-analysis. Journal of Sports Sciences.

[ref-15] Hidding LM, Chinapaw MJM, Belmon LS, Altenburg TM (2020). Co-creating a 24-hour movement behavior tool together with 9-12-year-old children using mixed-methods: MyDailyMoves. International Journal of Behavioral Nutrition and Physical Activity.

[ref-16] Hildebrand M, Hansen BH, Van Hees VT, Ekelund U (2017). Evaluation of raw acceleration sedentary thresholds in children and adults. Scandinavian Journal of Medicine & Science in Sports.

[ref-17] Hinkle DE, Wiersma W, Jurs SG (2003). Applied statistics for the behavioral sciences.

[ref-18] Kastelic K, Šarabon N, Burnard MD, Pedišić Ž (2022). Validity and reliability of the daily activity behaviours questionnaire (DABQ) for assessment of time spent in sleep, sedentary behaviour, and physical activity. International Journal of Environmental Research and Public Health.

[ref-19] Keadle SK, Patel S, Berrigan D, Christopher CN, Huang J, Saint-Maurice PF, Loftfield E, Matthews CE (2023). Validation of ACT24 version 2.0 for estimating behavioral domains, active and sedentary time. Medicine and Science in Sports and Exercise.

[ref-20] Lee J, Lee C, Min J, Kang D-W, Kim J-Y, Yang HI, Park J, Lee M-K, Lee M-Y, Park I, Jae SY, Jekal Y, Jee SH, Jeon JY (2020). Development of the Korean global physical activity questionnaire: reliability and validity study. Global Health Promotion.

[ref-21] Memon AR, Gupta CC, Crowther ME, Ferguson SA, Tuckwell GA, Vincent GE (2021). Sleep and physical activity in university students: a systematic review and meta-analysis. Sleep Medicine Reviews.

[ref-22] Migueles JH, Cadenas-Sanchez C, Ekelund U, Delisle Nyström C, Mora-Gonzalez J, Löf M, Labayen I, Ruiz JR, Ortega FB (2017). Accelerometer data collection and processing criteria to assess physical activity and other outcomes: a systematic review and practical considerations. Sports Medicine.

[ref-23] Migueles J, Rowlands A, Huber F, Sabia S, Van Hees V (2019). GGIR: a research community—driven open source R package for generating physical activity and sleep outcomes from multi-day raw accelerometer data. Journal for the Measurement of Physical Behaviour.

[ref-24] Nusser SM, Beyler NK, Welk GJ, Carriquiry AL, Fuller WA, King BM (2012). Modeling errors in physical activity recall data. Journal of Physical Activity and Health.

[ref-25] Peddie MC, Scott T, Haszard JJ (2021). Using a 24 h activity recall (STAR-24) to describe activity in adolescent boys in New Zealand: comparisons between a sample collected before, and a sample collected during the COVID-19 lockdown. International Journal of Environmental Research and Public Health.

[ref-26] Prince SA, Cardilli L, Reed JL, Saunders TJ, Kite C, Douillette K, Fournier K, Buckley JP (2020). A comparison of self-reported and device measured sedentary behaviour in adults: a systematic review and meta-analysis. International Journal of Behavioral Nutrition and Physical Activity.

[ref-27] Rollo S, Antsygina O, Tremblay MS (2020). The whole day matters: understanding 24-hour movement guideline adherence and relationships with health indicators across the lifespan. Journal of Sport and Health Science.

[ref-28] Rosenberger ME, Fulton JE, Buman MP, Troiano RP, Grandner MA, Buchner DM, Haskell WL (2019). The 24-hour activity cycle: a new paradigm for physical activity. Medicine and Science in Sports and Exercise.

[ref-29] Rowlands AV, Mirkes EM, Yates T, Clemes S, Davies M, Khunti K, Edwardson CL (2018). Accelerometer-assessed physical activity in epidemiology: are monitors equivalent?. Medicine and Science in Sports and Exercise.

[ref-30] Sabia S, Van Hees VT, Shipley MJ, Trenell MI, Hagger-Johnson G, Elbaz A, Kivimaki M, Singh-Manoux A (2014). Association between questionnaire- and accelerometer-assessed physical activity: the role of sociodemographic factors. American Journal of Epidemiology.

[ref-31] Sallis JF, Saelens BE (2000). Assessment of physical activity by self-report: status, limitations, and future directions. Research Quarterly for Exercise and Sport.

[ref-32] Song Y, Yoon YJ, Lee HJ, Kim YS, Spence JC, Jeon JY (2021). Development of a 24-hour movement behavior questionnaire for youth: process and reliability testing. Journal of Nutrition Education and Behavior.

[ref-33] Tremblay MS, Carson V, Chaput J-P, Gorber SC, Dinh T, Duggan M, Faulkner G, Gray CE, Gruber R, Janson K, Janssen I, Katzmarzyk PT, Kho ME, Latimer-Cheung AE, LeBlanc C, Okely AD, Olds T, Pate RR, Phillips A, Poitras VJ, Rodenburg S, Sampson M, Saunders TJ, Stone JA, Stratton G, Weiss SK, Zehr L (2016). Canadian 24-hour movement guidelines for children and youth: an integration of physical activity, sedentary behaviour, and sleep. Applied Physiology, Nutrition, and Metabolism.

[ref-34] Trost SG, McIver KL, Pate RR (2005). Conducting accelerometer-based activity assessments in field-based research. Medicine and Science in Sports and Exercise.

[ref-35] Vähä-Ypyä H, Vasankari T, Husu P, Suni J, Sievänen H (2015). A universal, accurate intensity-based classification of different physical activities using raw data of accelerometer. Clinical Physiology and Functional Imaging.

[ref-36] Van Hees VT, Sabia S, Anderson KN, Denton SJ, Oliver J, Catt M, Abell JG, Kivimäki M, Trenell MI, Singh-Manoux A (2015). A novel, open access method to assess sleep duration using a wrist-worn accelerometer. PLOS ONE.

[ref-37] Walsh JJ, Barnes JD, Cameron JD, Goldfield GS, Chaput J-P, Gunnell KE, Ledoux A-A, Zemek RL, Tremblay MS (2018). Associations between 24 h movement behaviours and global cognition in US children: a cross-sectional observational study. The Lancet Child & Adolescent Health.

[ref-38] Wang C, Chen P, Zhuang J (2013). Validity and reliability of international physical activity questionnaire-short form in chinese youth. Research Quarterly for Exercise and Sport.

[ref-39] Željko P, Adrian B (2015). Accelerometer-based measures in physical activity surveillance: current practices and issues. British Journal of Sports Medicine.

[ref-40] Zheng J, Tan TC, Zheng K, Huang T (2023). Development of a 24-hour movement behaviors questionnaire (24HMBQ) for Chinese college students: validity and reliability testing. BMC Public Health.

